# Global change ecology: Science to heal a damaged planet

**DOI:** 10.1371/journal.pbio.3002455

**Published:** 2023-12-11

**Authors:** Andrew J. Tanentzap, Olesya Kolmakova

**Affiliations:** 1 Ecosystems and Global Change Group, School of the Environment, Trent University, Peterborough, Ontario, Canada; 2 Ecosystems and Global Change Group, Department of Plant Sciences, University of Cambridge, Cambridge, United Kingdom

## Abstract

Addressing problems like climate change and food security required the advent of a new global-scale science. This Perspective reflects on the past 20 years of research in the field of global change ecology and looks at where the next 20 years might take us.

## Teaser

Humanity has drastically altered the biophysical systems that sustain life on Earth. We summarize progress and chart future directions in the emerging field of global change ecology, which studies interactions between organisms and their changing environment.

This article is part of the *PLOS Biology* 20th anniversary collection.

As we write, 2023 is set to be the warmest year on record. The year will end in the aftermath of one of the worst pandemics in modern history, rising food insecurity, and an accelerating extinction crisis. Georgina Mace foresaw that addressing these challenges would require a new global science [1], but what has this science achieved and where is it headed? To mark *PLOS Biology*’s 20th anniversary, we reflect on the past two decades of research in the field of global change ecology and where the next two decades might take us.

Global change ecology is a young field and has, until now, lacked a formal definition. To our knowledge, the term first appeared in a popular magazine article in 1991 [2] and gained wider attention in a 1994 review [3], neither of which included an explicit definition. Here, we broadly define global change ecology as the study of how human activities alter the interactions between populations or communities of organisms and the natural environment across large spatial or temporal scales. Major drivers of global change include climate change, land transformation, interference with biogeochemical cycles, pollution, and the transport of species beyond their native ranges [3]. The field has its roots in early ecology that aimed to estimate the flow of energy and elements through ecosystems and the primary production of the Earth as a whole [4]. Independently, the application of systems science to study how different parts of the natural environment, such as the biosphere or atmosphere, interact with each other has laid the foundation for understanding human impacts on these systems and their interactions [5]. Arguably synonymous with the term global change biology that studies the responses of species to environmental change, global change ecology focuses on the interactions between organisms and their changing environments, such as feedbacks onto biophysical processes.

Only 3 years after the first issue of *PLOS Biology* was published, William Schlesinger published the first review of global change ecology [4]. Revisiting the research undertaken thereafter, we think there is reason to be hopeful that we can tackle the societal challenges that global change ecology aims to solve (Table 1).

**Table 1:** Revisiting progress and knowledge gaps in answering Schlesinger’s [4] priorities for global change ecology.

Original question	What we now know	What remains unknown
What are the sources of biogenic elements in the ocean?	Anthropogenic deposition is a major transport pathway for reactive nitrogen and iron. Increased ocean stratification is reducing upwelling and trapping nutrients in deeper layers.	Relative importance of anthropogenic dust deposition towards elemental budgets.
Are major sources of toxic pollutants natural or anthropogenic?	Pollutants like mercury and persistent organic pollutants are largely anthropogenic and declining due to global treaties.	At what concentrations do emerging contaminants (e.g., microplastics, per- and polyfluoroalkyl substances) disrupt biophysical processes?
What proportion of species are functionally redundant?	Most species contribute unique functions because of spatial and temporal niche differentiation.	The specific functions of species in different contexts, including their contribution towards ecosystem stability.
How do genetically modified organisms impact biodiversity?	Gene flow and integration of transgenes occurs into wild populations.	Whether transgenes and hybrids outcompete wild species, although this is likely a far smaller threat to biodiversity than habitat destruction and climate change.
What are the impacts of biotic homogenization?	Reduces multifunctionality at landscape scales [6].	Impact on ecosystem resilience to future perturbations.
How will humanity respond to pandemics?	Incredibly poorly in terms of social and political responses, despite the best efforts of healthcare workers and scientists sharing knowledge and developing vaccines and treatments.	How will humanity respond to the next pandemic?
Is our lifestyle sustainable?	No country meets the social needs of its citizens with a sustainable use of natural resources [7].	What is the contribution of climate change mitigation and adaptation initiatives? How can the Sustainable Development Goals best be implemented?

The Sustainable Development Goals, adopted by the United Nations General Assembly in 2015, have especially given “reality and meaning” to efforts to protect the environment while meeting human needs: one of Schlesinger’s [4] priorities for global change ecology. A key milestone in translating science into policy has also been increasing public acceptance that global change, including climate warming, is caused mostly by human activities [8]. Changes in public attitudes have emerged alongside the growing reputation of the Intergovernmental Panel on Climate Change and the younger Intergovernmental Science-Policy Platform on Biodiversity and Ecosystem Services. In the past 20 years, both of these bodies have been at the forefront of synthesizing scalable solutions to tackle global change and moved beyond simply proving that humans cause widespread planetary change. New technologies, such as biophotovoltaics to generate clean energy [9], and large-scale restoration strategies, such as rewilding [10], have further emerged to help reverse humanity’s impact on the planet.

Alongside the need for sustainable development, Schlesinger [4] identified two other priorities in global change ecology, for which considerable progress has been made in the past 20 years (Table 1). The first priority pertains to the 70% of our planet covered by the oceans. We increasingly understand how essential elements such as nitrogen, phosphorus, and iron fuel marine primary productivity and how they might be manipulated to address problems like climate change, such as by increasing carbon storage [11]. Likewise, we have considerably improved our understanding of the sources of toxic pollutants in the oceans, enabling us to identify interventions that reduce their transport and formation. For example, the finding that atmospheric deposition is responsible for most of the mercury in the world’s oceans led to the 2013 Minamata Convention on Mercury that aims to reduce anthropogenic mercury emissions. Our knowledge has also expanded to include pollutants such as microplastics and per- and poly-fluorinated substances, which were virtually unstudied 20 years ago. One downside of this attention on the oceans is that it has arguably overshadowed freshwaters, which are disproportionately important for Earth system processes and human wellbeing relative to the land that they occupy. Thus, understanding how to protect and restore freshwaters, especially through their connections with surrounding landscapes, should be a priority for the next 20 years of global change ecology.

The final priority that Schlesinger [4] identified was understanding the role of species in delivering functioning ecosystems. We now know that the role of each species in a community is highly context-dependent, so conserving the largest diversity of species is best to maintain different processes within an ecosystem [12]. Furthermore, we understand more about how the global redistribution of species by people has shaped ecological processes at large spatial scales [12]. Major achievements have especially arisen in our understanding of microorganisms. Microbes constitute the overwhelming majority of life on Earth, both in terms of diversity and sheer numbers of individuals, and are essential to all biophysical processes that sustain living organisms. Yet 20 years ago, the microbiome was largely a “black box”; we knew very little about its internal workings. The “omics” revolution, along with global scientific initiatives like the *Tara* Oceans project, have generated new understanding of how microbes sustain the global climate and biogeochemical cycles [13], moving biodiversity research beyond just counting species. Breakthrough technologies, including real-time environmental DNA sequencing, autonomous vehicles, remote sensing, and artificial intelligence, further promise to accelerate our understanding of the role that species have in the Earth system and how they will be impacted by human activities.

The next 20 years of *PLOS Biology* will coincide with one of the most tumultuous times in humanity’s existence on Earth. It is more likely than not that global mean temperatures will be 1.5°C warmer than pre-industrial levels within just a few years, with pollution and habitat destruction continuing unabated. Although not intended to be exhaustive, we envision three interconnected areas where global change ecology can make considerable progress.

First, a major question will be how to reduce the environmental impacts of global change adaptation and mitigation strategies. For example, increasing extraction of critical minerals to build much of our climate change mitigation technology, such as solar panels and lithium-ion batteries, risks further damaging the natural environment. Natural climate solutions, such as large-scale tree planting and ocean iron fertilization, also have many negative effects [14]. Implementing adaptation and mitigation in ways that co-benefit social and environmental outcomes remains a major obstacle for sustainable development [5].

A related question is how will global change shape the interactions among biophysical systems at the planetary scale. Again, this knowledge is necessary for effective global change adaptation and mitigation, as well as for efforts to model the Earth system [5]. For example, if increasing pollution and ocean acidification kill most marine plankton, the ocean’s ability to sequester atmospheric carbon could be drastically reduced. This interaction could turn into a positive feedback given that atmospheric carbon dioxide is the primary cause of ocean acidification.

Finally, an important question is how is global change rewiring interactions among species. New opportunities afforded by the study of ancient environmental DNA will allow us to track these interactions over the geological timescales on which they developed, including through large-scale climate oscillations, so as to inform us of how these interactions will adapt in the future. This question also includes the emergence of novel pathogens from a rapidly thawing cryosphere and human encroachment into the planet’s remaining wild habitats. One Health is a key concept that will guide this question. The concept has emerged within the past 20 years to recognize that the health of people, animals, and the environment are closely interconnected, thereby embracing the need for transdisciplinary approaches that unite natural and social sciences. Transdisciplinary approaches are also emerging as mandatory for effective biodiversity conservation, which largely involves managing people rather than nature. Indigenous knowledge has an important role here, often providing generations of lived experience for how to coexist sustainably with the environment. We hope that the next 20 years of *PLOS Biology* will be filled with many such inspiring solutions to the challenges confronted by global change ecology.
